# Design of an impact evaluation using a mixed methods model – an explanatory assessment of the effects of results-based financing mechanisms on maternal healthcare services in Malawi

**DOI:** 10.1186/1472-6963-14-180

**Published:** 2014-04-22

**Authors:** Stephan Brenner, Adamson S Muula, Paul Jacob Robyn, Till Bärnighausen, Malabika Sarker, Don P Mathanga, Thomas Bossert, Manuela De Allegri

**Affiliations:** 1 Institute of Public Health, Ruprecht-Karls-University, Heidelberg, Germany; 2 Department of Community Health, University of Malawi, College of Medicine, Blantyre, Malawi; 3 The World Bank, Washington, DC, USA; 4 Department of Global Health and Population, Harvard School of Public Health, Boston, Massachusetts, United States of America; 5 Wellcome Trust Africa Centre for Health and Population Studies, University of KwaZulu-Natal, Mtubatuba, South Africa

**Keywords:** Mixed methods, Impact evaluation, Performance-based incentives, Study design

## Abstract

**Background:**

In this article we present a study design to evaluate the causal impact of providing supply-side performance-based financing incentives in combination with a demand-side cash transfer component on equitable access to and quality of maternal and neonatal healthcare services. This intervention is introduced to selected emergency obstetric care facilities and catchment area populations in four districts in Malawi. We here describe and discuss our study protocol with regard to the research aims, the local implementation context, and our rationale for selecting a mixed methods explanatory design with a quasi-experimental quantitative component.

**Design:**

The quantitative research component consists of a controlled pre- and post-test design with multiple post-test measurements. This allows us to quantitatively measure ‘equitable access to healthcare services’ at the community level and ‘healthcare quality’ at the health facility level. Guided by a theoretical framework of causal relationships, we determined a number of input, process, and output indicators to evaluate both intended and unintended effects of the intervention. Overall causal impact estimates will result from a difference-in-difference analysis comparing selected indicators across intervention and control facilities/catchment populations over time.

To further explain heterogeneity of quantitatively observed effects and to understand the experiential dimensions of financial incentives on clients and providers, we designed a qualitative component in line with the overall explanatory mixed methods approach. This component consists of in-depth interviews and focus group discussions with providers, service user, non-users, and policy stakeholders. In this explanatory design comprehensive understanding of expected and unexpected effects of the intervention on both access and quality will emerge through careful triangulation at two levels: across multiple quantitative elements and across quantitative and qualitative elements.

**Discussion:**

Combining a traditional quasi-experimental controlled pre- and post-test design with an explanatory mixed methods model permits an additional assessment of organizational and behavioral changes affecting complex processes. Through this impact evaluation approach, our design will not only create robust evidence measures for the outcome of interest, but also generate insights on how and why the investigated interventions produce certain intended and unintended effects and allows for a more in-depth evaluation approach.

## Background

The strategic purchasing of healthcare services, together with the generation of sufficient financial resources for health and adequate risk pooling mechanisms, represents an essential function of any health care financing system [1]. Otherwise defined as “financing of the supply side”, the purchasing function determines what services are bought, in what quantity, for whom, from which providers, and according to what payment modalities [2]. In general, purchasing arrangements are expected to set the right incentives for providers to deliver the adequate amount of quality healthcare services to all those entitled to receive care within existing coverage conditions [3].

In low- and middle-income countries (LMICs), health care purchasing models traditionally comprise mixtures of direct or indirect input-based arrangements (i.e. salaries, commodities, capital investments) directly covered and managed by national governments, and direct output-based fee for service payments (i.e. formal and informal user fees) [4,5]. Health care purchasing models in which public sector providers heavily rely on input-based financing do not create strong enough incentives to deliver the sufficient quantity and/or quality of services. These models have long been proven to suffer from a number of flaws and inefficiencies, including high rates of provider absenteeism, poor quality of service delivery, and frequent drug shortages [6-8]. In addition, the system’s extreme dependency on direct user payments shifts the main responsibility to cover healthcare costs to the ill at point of service use. Especially in LMICs, the combination of weak provider incentives and high fee for service user payments further enhances already existing gaps in service coverage by making care inaccessible to many communities [9].

In response to the alarming gaps in coverage and quality of care observed across LMICs, a new set of purchasing models has emerged as a potential alternative to traditional input-based financing arrangements. A common feature underlying these new purchasing models is the focus on health service outputs. Encompassing a variety of implementation experiences, outputs – defined in terms of quantity and/or quality of services delivered – function as the basis against which health authorities determine and authorize provider payments. As such, these output-based financing arrangements are commonly labelled Results-Based Financing (RBF) or Performance-Based Incentives (PBI) [10]. Such PBI aim at counteracting the flaws of input-based financing (and when coupled with additional complementary interventions also the flaws of user fees) by steering the purchasing function, so that providers are incentivized to provide the high-quality service outputs necessary to meet the community’s health care needs.

Traditional input-based forms of healthcare financing tend to invest relatively large amounts of funds into numerous healthcare input factors, such as health facility infrastructure, healthcare personnel, technical equipment and supplies. In contrast, PBI strategies tie financial incentives directly to the expected healthcare outputs. To do so, PBI models introduce contractual frameworks that define not only the roles and responsibilities of purchasers and providers, but also clearly outline the output targets and output-dependent incentives, the result verification processes confirming the delivery of such outputs, and the payment mechanisms in response to the obtained results [11].

In LMICs, PBI have been introduced to improve the quality of the services delivered and to increase health service utilization. Most PBI strategies have a service quality focus and incentivize healthcare providers to adhere to clinical standards, to participate in training and accreditation programs, or to respect patient-centeredness, which all are considered pathways of supply-driven service utilization leading to improved health outcomes [12]. Besides the targeting of providers, in some instances, PBI have also been used to counteract health service under-utilization through specific targeting of client demand and service availability [13].

Two common forms of PBI are Performance-Based Financing (PBF) and Conditional Cash Transfers (CCT). By definition, PBF programs target the supply and delivery of healthcare services by incentivizing healthcare providers, either as individuals or in form of entire health facilities. Financial rewards are usually paid in form of salary top-ups based on fee-for-service payments in relation to quality service outputs [10,14,15]. CCT programs target demand for or utilization of healthcare services. Their beneficiaries are healthcare users who are incentivized to enroll into specific health programs or to comply with certain health-related behaviors. Direct financial payments to users are thus related to the degree of compliance [10,16].

Although a promising feature in public sector health service regulation, there is only limited evidence of the effectiveness of PBI programs on healthcare outcomes in Sub-Saharan Africa. Since PBI programs incentivize mainly quantity or quality of healthcare outputs, effects on healthcare outcomes are more difficult to capture and depend on how predictive an output measure is for an expected outcome measure [17]. As healthcare outcomes are only indirectly linked to what can be directly influenced by a single provider’s performance or service user’s behavior, studies analyzing the impact of PBI programs focus on service output measures related to utilization rates or number of cases treated. With regard to maternal and child healthcare service outputs, evidence from PBF pilots demonstrated that introduction of financial incentives based on provider performance improves healthcare quality, increases service utilization, creates more efficient financial and organizational management structures, and restricts corruption [18-21]. In other instances, some evidence is available to show that PBF programs further contribute to inequalities in access, encourage healthcare providers to adopt ‘gaming’ behaviors, lead to neglect of non-incentivized healthcare services, or are too cost-intensive in terms of their long-term sustainability [22,23]. In addition, there is still limited understanding on the effects of performance-based incentives on the intrinsic and extrinsic motivation of healthcare providers [24,25]. Given the variety in PBI implementation and evaluation, strong evidence on the impact of PBI programs on both healthcare outputs and outcomes in LMICs remains extremely meager [26,27].

As outlined above, a vast majority of recent PBI impact evaluations in LMICs have been based on purely quantitative research designs. In light of the current need for stronger evidence on the relationship between PBI and health outcomes, especially in sub-Saharan African countries, the purpose of this article is to describe a rigorous mixed methods research protocol designed to evaluate the causal impact of a PBI program currently rolled out in Malawi on health service structures, processes, and outputs. The study design uses a sequential explanatory mixed methods approach developed to assess the causal relationship between a set of PBF and CCT incentives and a number of maternal health service outcomes. In the following sections, we outline the PBI program’s implementation context and its influence on our impact evaluation design. We then further describe the overall study design by illustrating each of the proposed study components. We also define the rationale and purpose behind each component as they relate to the current PBI evidence gaps. Finally, we discuss the advantages of an explanatory mixed methods design in addressing a number of essential characteristics in the evaluation process of a health system intervention. With the strategic application of quantitative and qualitative methods, as in our suggested research design, we provide an example of an impact evaluation approach that allows a broad evaluation focus with a high yield of robust measurements of the underlying treatment effect. In sharing this protocol we aim to provide an example of how qualitative methods can be integrated into commonly used quantitative impact evaluation designs.

## Design

### Study setting

Malawi, like many sub-Saharan countries, is not on track to meet its targets for Millennium Development Goal five. In 2010, Malawi’s maternal mortality ratio (675/100,000 live births) and neonatal mortality rate (31/1,000 live births) were among the highest in the world [28]. Poor managerial and organizational quality of care together with lack of adequate resources is largely responsible for delays in care which ultimately result in the death of women and their newborns [29]. The Essential Health Package introduced in 2004 lists maternal care among those health services that should be provided free of charge in Malawi, which led to an increase in maternal health service coverage and utilization in the following years [30,31]. In 2010, 46% pregnant women were found to attend at least four antenatal care visits and 71% pregnant women delivered in the presence of a skilled attendant [28]. In spite of these relatively high coverage rates, quality of care deficits due to persistent financial and geographical barriers, ongoing deficiencies in human resources for health, and frequent stock-outs of essential equipment and drugs still contribute to poor maternal health outcomes [26].

With the ultimate objective of reducing maternal and neonatal mortality, the *RBF4MNH Initiative* was introduced by the Ministry of Health (MoH) of Malawi with financial support of the Norwegian and German governments. Options Consultancy Services was contracted by the MoH for technical support in implementing and monitoring the project. The RBF4MNH Initiative seeks to improve quality of maternal and neonatal healthcare (MNHC) delivery and utilization in public and private not-for-profit health facilities [32]. The Initiative’s primary objective is to increase the number of deliveries that take place under skilled attendance in district-level and rural health facilities with high quality maternal and neonatal services provision. For this purpose, the Initiative currently targets 17 emergency obstetric and neonatal care (EmONC) facilities, of which 13 operate at the basic (rural health centers) and four at the comprehensive (4 district hospitals) level. These 17 facilities were selected out of all 33 facilities that are supposed to provide EmONC services according to WHO service coverage criteria (i.e. one comprehensive and four basic EmONC facilities per 500,000 population [33]) as identified by the MoH within four districts (Balaka, Dedza, Mchinji, and Ntcheu). Facility selection was carried out jointly by the District Health Management Teams (DHMT), the head of the Reproductive Health Unit (RHU) of the MoH and the Options team. In a first step, a broad quality assessment was conducted based on a number of performance indicators related to four main health service functions: a) leadership, b) resource management, c) environmental safety, and d) service provision. In a second step, only those facilities with maternal care services performing all required EmONC signal functions, operating day and night, having at least three qualified staff in place, meeting WHO-recommended population coverage criteria, and having a functional referral system in place were selected into the intervention.

Programmatically the RBF4MNH Initiative is divided into three major components: a) a basic *infrastructural upgrade* of the 17 facilities including architectural modification to extend available space, replacement and provision of essential equipment, and maintenance of critical supply chains necessary in sustaining EmONC minimum standards; b) a *supply-side PBF intervention* consisting of quality-based performance agreements between the RHU on the one side and targeted facilities and DHMT on the other; and c) a *demand-side CCT intervention* consisting of monetary compensations to pregnant women for the recovery of expenses directly related to accessing and staying at target facilities during and at least 48 hours after childbirth.

The initial phase of the infrastructural upgrade component was completed prior to the official introduction of the PBF and CCT program components, but for some facilities still ongoing due to unforeseen administrative and logistic delays. In early April 2013 performance agreements were signed between the MoH, the 17 health facilities, and the four respective DHMT. Within these agreements, performance rewards are linked to quantity and quality indicators. All indicators are directly or indirectly related to the Initiative’s primary outcome to increase the number of hospital-based deliveries of good quality and can be divided into two groups: a) *core indicators* to determine health facility and DHMT reward payments based on the achievement of set targets; and b) *quality indicators* to deflate reward payments based on deficits in performance quality. The core indicators measure among others the quantity of facility-based deliveries at BEmOC level facilities, HIV screening tests offered, PMTCT treatments provided, maternal death audits conducted, sufficient stocking of necessary medicines, and the timely completion and submission of HMIS reports. The quality indicators measure quality aspects of technical care during labor, delivery, and newborn care provided (e.g. use of partograph during first stage of labor, use of oxytocics drugs during third stage of labor, use of magnesium sulfate in cases of (pre-)eclampsia, supplementation of vitamin A to newborns), but also assess the level of provider adherence to routine service processes (e.g. patient feed-back mechanisms, equipment repair protocols, infection control guidelines). Indicators receive different weights that are used in the calculation of financial rewards.

Within the agreements, performance targets for each facility and DHMT are set individually. Performance reports on core and quality indicators are submitted by each health facility and DHMT to the RHU, and reported data is afterwards verified by an external verification agent. The first verification was organized as peer review of districts to facilitate joint learning. Based on the verified results and in relation to the achievement of the targets the entire staff (maternity unit plus other clinical units) of district hospitals (CEmONC), the entire staff at health centers (BEmONC), and the DHMTs are rewarded. These financial rewards are earmarked in a way to ensure both facility investments (30%) and salary top-ups (70%), which average 15–25% of health staff’s total salary envelope. Facilities are free to use the facility portion of the rewards to finance any infrastructural improvements independent of direct relevance to MNHC delivery. The rewards of the DHMTs are not only based on the achievements of the selected health facilities but also on the achievements of the district as a whole in order to avoid that DHMT support is targeted towards single facilities. Verification and payment cycles are scheduled to occur every six months.

Concomitantly with the supply-side rewarding scheme a demand-side scheme was introduced in July 2013. The demand-side intervention consists of CCTs targeted towards pregnant women living in the EmONC catchment areas of the 17 selected facilities. The CCTs are intended to support women a) to present to the intervention facilities for delivery in time; and b) to remain under skilled maternal care observation at these facilities for the initial 48-hour post-partum period. The financial support is considered as cash contributions towards costs incurred by delivering in a health facility, such as expenses related to transport to and from the facility, food while staying at the facility, and essential childbirth items (blankets, wrapping cloth). Enrollment into the CCT scheme occurs during a woman’s first antenatal care visit at the respective health facility. Upon enrollment, all eligible women (i.e. permanent residence in the EmONC catchment area) are given a *cash transfer card* to keep with them until the day of delivery. Following initial enrollment, Health Surveillance Assistants (HSA) at the community level will verify each woman’s eligibility based on her residential status. Upon delivery at a target facility, all enrolled women are given a cash amount consisting of: a) a fixed amount to cover essential childbirth expenses; b) a variable amount covering transport expenses based on the actual distance between health facility and a woman’s residence; and c) a fixed amount for each 24 hours up to a total of 48 hours following childbirth a women stays under clinical observation at the facility to cover food and opportunity costs (loss of productivity being away from home).

The PBF supply-side incentives and demand-side CCTs are expected to increase the number of facility-based deliveries through their combined effect on improved quality, through changes in providers’ motivation and proactivity, and the removal of financial barriers to access. Accordingly, any observed changes in institutional delivery rates – from an implementation point of view – can be directly linked to positive improvements in current MNHC service delivery and service utilization (i.e. program outputs).

### Research objectives

The impact evaluation design presented in this article was developed by a multi-institutional team of researchers. As this design is used for an external evaluation process, all researchers are independent of the MoH and its implementation team. This impact evaluation study is independently funded by USAID/TRAction and by the Norwegian Government. The evaluation study was conceptualized to assess the impact of the RBF4MNH Initiative for a period of approximately 24 months after implementation.

Given the relatively short assessment period, the size of the study population, and the resulting power to detect a reasonable effect size, it is not feasible for our evaluation design to use population-based indicators, such as maternal mortality ratios and neonatal mortality rates, as outcome measures. Such long-term indicators are not sensitive enough to capture the expected PBI effects, especially since the results incentivized by the RBF4MNH Initiative directly target health care outputs, not outcomes or impacts. Our focus remains therefore on short- and mid-term structural, procedural, and output measures to sufficiently capture any intermediate effects resulting from the PBI intervention, which are all understood to contribute to the reduction of maternal and neonatal mortality. The main study objectives are to evaluate the impact of the RBF4MNH Initiative on measures of quantity and quality related to the delivery and utilization of maternal and neonatal healthcare services. For this purpose, we chose quality of care and access to care as outputs of interest as they are closely related to maternal and neonatal health outcomes, such as maternal and neonatal mortality, morbidity, and disability [34,35]. Furthermore, both service quality and service accessibility are under direct influence of the health system [36].

In the light of the current PBI evidence gap in Sub-Saharan Africa, we also follow a number of additional research objectives that more directly address the current scientific discussion on performance incentives in health financing. First of all, we introduce e an assessment of the extent of negative PBI effects on the delivery of health services not directly targeted by financial incentives. For this we broaden the study focus beyond structure, process and output measures related to obstetric care only to also include other health services along the continuum of maternal care, namely antenatal care (ANC) and postnatal care (PNC) services. Second, since research on provider motivation is still limited, we included an assessment of the interaction between financial incentives and provider behavior based on experiential accounts of the working environment in response to a PBF package. Last, as the RBF4MNH Initiative offers a CCT package independent of any specific pro-poor targeting strategy, we also consider an assessment of the distributional impact among clients of different socioeconomic backgrounds as relevant.

Based on the main study objectives and in conjunction with the additional research objectives, we defined the following specific research aims:

• Specific research aim 1: *To establish the effect of supply-side and demand-side incentives on quality of health care services in Malawi.*

  It is our hypothesis that the RBF4MNH supply-side incentives to maternal care providers and DHMTs have a positive effect on the quality of obstetric services. We anticipate that the extent to which the performance-based incentives create expected changes in service quality will depend, positively or negatively, on the level of service utilization produced by the demand-side incentives. We expect that those districts where supply and demand of quality of care is met most optimally will demonstrate the most pronounced outcome measures.

• Specific research aim 2: *To establish the effect of supply-side and demand-side incentives on the utilization of maternal healthcare services in Malawi.*

  It is our hypothesis that the demand-side incentives to pregnant women have a positive effect on the utilization of facility-based obstetric care services. We anticipate that the extent to which the CCT incentivizes change health-seeking behaviors of women will be more pronounced in districts where access barriers are relatively high. We also expect that in those districts where improvements in quality of service delivery in response to the supply-side incentives are most successful outcome measures for service utilization will be most pronounced.

• Specific research aim 3: *To establish the effect of supply-side and demand-side incentives on the access to and quality of not directly incentivized maternal care services.*

  It is our hypothesis that the strong focus on incentivizing quality of care and utilization of obstetric services will not affect the quality of other maternal care services to a significant extent. We anticipate that only those aspects of care similar to all three services, such as stocking of essential medicines or timely submitted HMIS reports, might have some impact on the quality of ANC and PNC services. Within obstetric care service delivery processes we expect some providers (least satisfied, least trained) to focus mainly on those activities directly related to incentivized outputs.

• Specific research aim 4: *To establish the effect of supply-side and demand-side incentives on the experiential dimensions of healthcare quality among maternal care providers and clients.*

  It is our hypothesis that the combination of supply-side and demand-side incentives will generate different varieties of reactions and responses based on individuals’ experiences with incentives and reward systems. We anticipate positive as well as negative individual experiences of providers with performance incentives with regard to workload, job satisfaction, or motivation. We also expect similarly broad experiences of clients with CCT incentives in relation to changes in perceived quality of care, service accessibility, or health-seeking behavior.

• Specific research aim 5: *To establish the extent to which the demand-side incentives generate equitable access to care for pregnant women.*

  It is our hypothesis that the CCTs will increase utilization of obstetric services for pregnant women currently facing financial access barriers, and thus result in more equitable utilization. Still, we expect persisting additional barriers, financial and non-financial, to prevent some women from enrollment into the CCT scheme prior to childbirth.

### Conceptual framework

**Figure 1:**
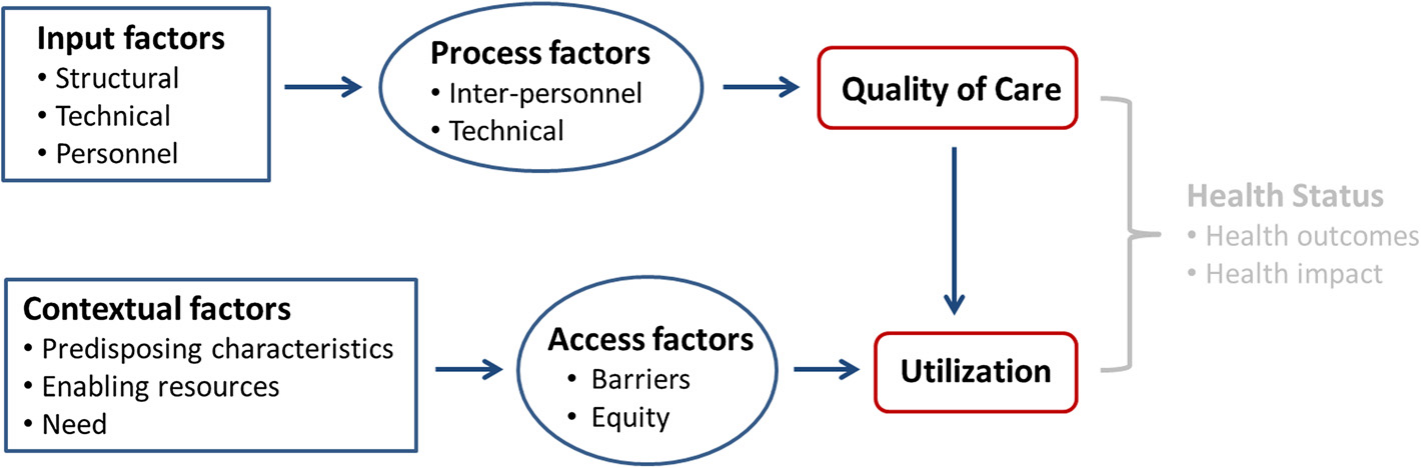
Conceptual framework of quality of care and utilization.

Both quality of care and service utilization are challenging to assess since each one of them represents a complex theoretical construct. For our impact evaluation approach, we chose the following conceptual models to allow for a comprehensive assessment of the expected impact of the intervention on quality and utilization patterns. The resulting conceptual framework underlying our evaluation study is illustrated in Figure 1.

• *Quality of care*, according to the Donabedian model [37], results from a sequence of three elementary steps. First, a *structural element* comprising service-related technical and human input factors; second, a *process element* comprising technical and interpersonal activities needed to transform structural elements into actual healthcare outputs; and third, an *output element* comprising health service products generated by the input and process elements. Based on this model, healthcare quality can be defined as an outcome product that is dependent on both sufficient input and efficient process factors. An additional qualitative approach to this model will serve to elucidate the experiential dimensions of service delivery, understanding how care is delivered and why [38]. In particular, the qualitative element will explore the social and cultural setting of service delivery, shedding light on why providers manage the clinical encounter the way they do, what are facilitating and hindering elements to the delivery of quality care (within and beyond the PBF intervention), and what elements are responsible for motivation and satisfaction (within and beyond the PBF intervention).

• To frame *healthcare utilization,* we adapted Andersen’s *behavioral model of health services use*[39]. Based on this model, health care utilization results from determinants of access. Access to care is in turn further defined along a number of contextual and individual characteristics: a) *predisposing characteristics* such as demographic and social structures, individual health beliefs, or the role of a sick person within a community; b) *enabling characteristics* such as income, insurance coverage, user fees, travel and waiting times; and c) *need characteristics* such as the perceived urgency or prior experience with a given health problem. In this model, *access* is a prerequisite for healthcare utilization. Access is understood as *equitable* when healthcare utilization solely depends on individual need irrespective of other factors such as age, sex, income, or ethnicity [40].

### Theory of change

**Figure 2:**
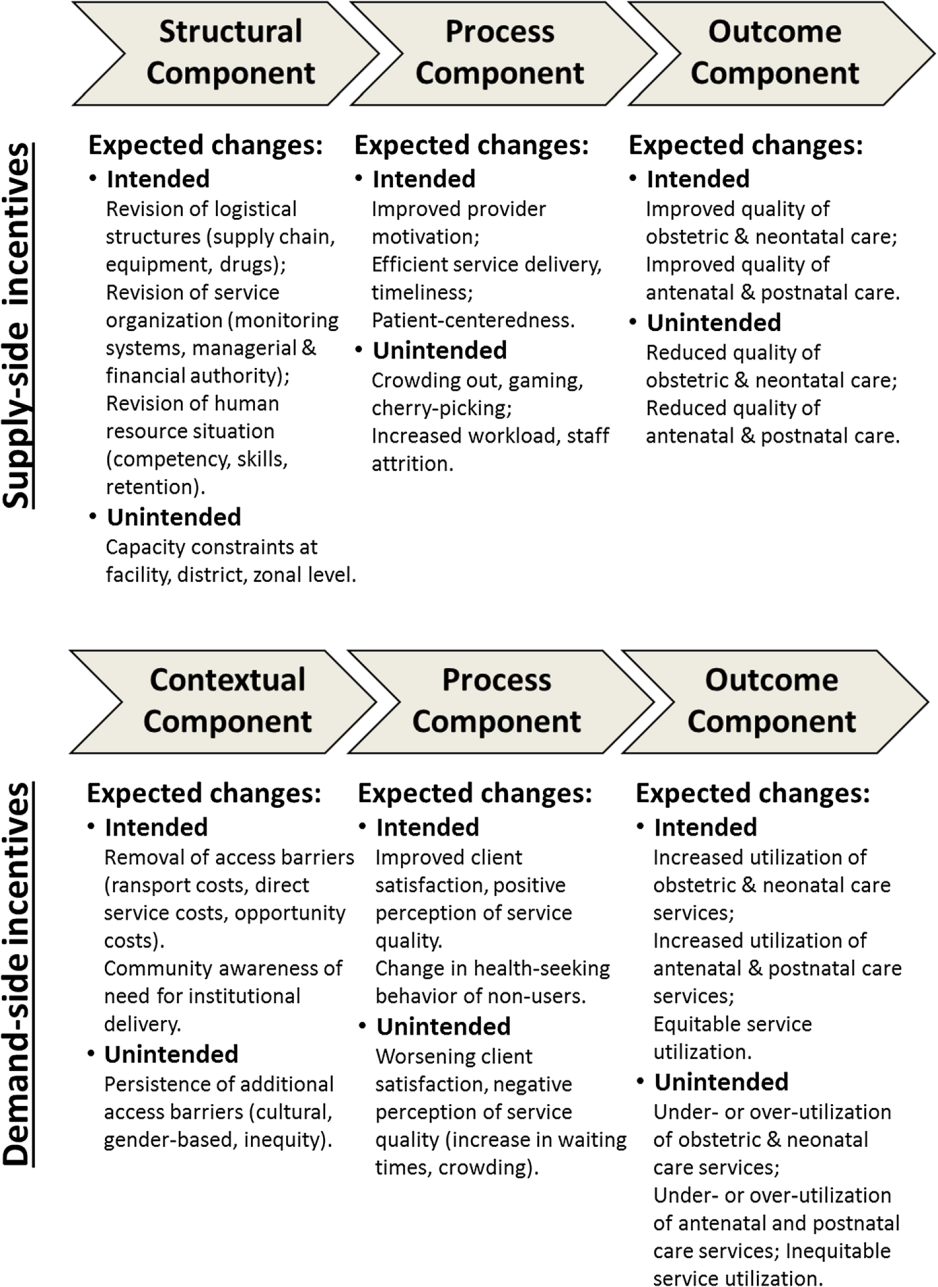
Causal chains addressed by the impact evaluation design.

Guided by the research aims and based on the selected conceptual framework, we used our hypotheses about the cause-effect relationships to further outline a comprehensive theory of change. As shown in Figure 2, the theory of change allows us to map the causal chains between supply- and demand-side incentives and to identify some of the expected intended and unintended effects.

The supply-side incentives affect both the structural and procedural elements of healthcare quality positively. Following this causal chain of events, providers’ autonomy, motivation and satisfaction increase. Ultimately, improved effectiveness, more timeliness, and higher quality of EmONC service delivery result. Since these supply-side incentives only target quality outputs of obstetric care services, the cause-effect relationship can play out in two possible ways. Either with the effect of positive quality outcomes not only in obstetric but also in the delivery of ANC and PNC services, as is imaginable in rural health facilities where the entire continuum of MCH is provided by the same cadre of health professionals, or with the effect that the focus on obstetric care only leads to neglect of service quality of the non-incentivized ANC and PNC services. Alternatively, the supply-side incentives carry the risk to crowed out providers’ intrinsic motivation. In this causal chain the loss of altruistic behavior and work ethics with active manipulation of the rewarding system for personal gain does ultimately not yield any positive changes in service quality outcomes.

In addition, the demand-side incentives remove financial access barriers and potentially allow more pregnant women to utilize obstetric services. Following this causal chain of events, individual and household expenditures related to facility-based deliveries are lowered as expenditures associated with transport, childbirth equipment, and opportunity costs are compensated. Furthermore, since enrollment of women into the CCT program is organized through ANC visits, the number of pregnant women who attend ANC services increases. Provided quality of care is perceived as high, as more women deliver at health facilities, the utilization of PNC and other services within the MCH continuum rises as a result of patient education and trust in established provider-patient relationships. Since these demand- and supply-side incentives are applied independently of each other the effects on utilization might exceed the capacity in quality service delivery. In this causal chain, the demand-side incentives affect pregnant women’s health-seeking behavior to the extent that they not only stay the intended 48-hours postpartum at the facility, but also present days prior to labor onset to the maternity services. As facilities’ capacity in maternal waiting homes, maternity beds, and midwives is limited, service over-utilization is likely to result, which over time can lead to negative implications on the level of quality provided. Alternatively, if the perception pregnant women have of service quality remains low, removal of financial access barriers alone are not necessarily sufficient enough to increase utilization. Following this causal chain, little or even no change in utilization of facility-based obstetric care services is a likely consequence.

### Mixed methods study design

**Figure 3:**
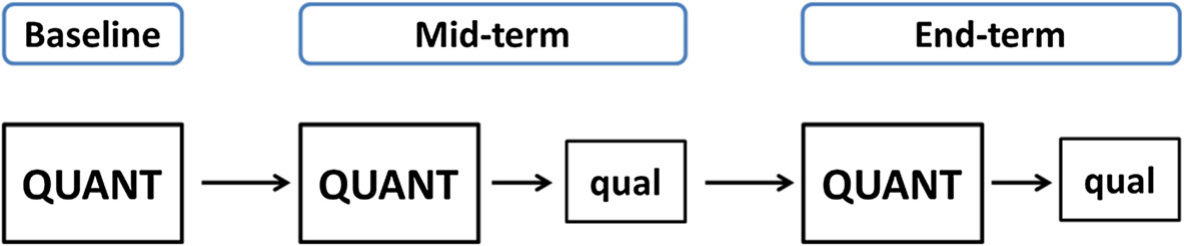
**Sequential explanatory mixed methods design.** QUANT = dominant quantitative study component, qual = sequential qualitative study component.

The methodological framework of our impact evaluation follows an *explanatory mixed method design*. In mixed methods research, ‘explanatory’ describes the purposeful inclusion of qualitative methods of data collection and analysis to “explain” the quantitative results [41]. In an explanatory mixed methods design, quantitative research components dominate over qualitative ones. This fact makes explanatory models very suitable for impact evaluations, as impact measures are usually of quantitative nature. Nevertheless, analyzing quantitative data in the presence of qualitative information supplies additional input for the interpretation of overall results. In an explanatory mixed methods approach therefore, sequencing quantitative by qualitative research components provides additional information on unexpected or unexplainable results. In line with the rationale of an explanatory mixed methods design in our study, qualitative data collection follows quantitative data collection at mid-term and endpoint. Given that the focus of the qualitative work is on “explaining” the quantitatively measured changes produced by the intervention, there is no need for a qualitative data collection at baseline, meaning before the intervention has even started. Figure 3 schematically displays the anticipated sequences of the research components within our explanatory mixed methods design. The rationale behind selecting an explanatory mixed methods design is guided by our research aims. Using quantitative and qualitative methods sequentially to explain study results allows us: a) to comprehensively capture the complexity of the impact measures (i.e. quality of care, utilization); b) to keep a broader scientific scope to investigate on intended and unintended effects; and c) to yield sufficient credibility and validity of the resulting impact estimates. In addition, the qualitative information will be particularly helpful to illuminate the heterogeneity in effects we expect to observe across facilities, communities, and households. A better understanding of relevant contextual elements facilitating or hindering change yields valuable information, as it allows unraveling under which conditions PBI schemes can be expected to produce which results.

As an explanatory mixed methods design relies heavily on the robustness of quantitative data, the set-up of the quantitative research component is a crucial factor. For the purpose of our impact evaluation, we structured the quantitative research component based on a quasi-experimental design [42] in the form of a *controlled pre- and post-test design* with two post-test measurements. This allows us to collect data at baseline (prior to the implementation of incentives), at mid-term (approximately one year after the incentives are in place), and at end-point (towards the end of the impact evaluation funding period). Our quantitative design is ‘controlled’ since we collect and compare data from both intervention sites (i.e. RBF4MNH-targeted EmONC facilities and EmONC catchment areas) and control sites (i.e. non-targeted EmONC facilities and corresponding EmONC catchment areas) during each of the three data collection rounds. As described earlier, of the 33 facilities authorized to provide EmONC in the concerned districts, 17 were non-randomly included in the RBF4MNH intervention. The remaining 16 facilities are chosen as control sites for the impact evaluation study. The rationale behind using a controlled pre- and post-test design to assess quantitative effects is in response to the fact that randomization was not possible due to implementation considerations. As for the rationale behind the selection of the control sites, the implementation process followed by the RBF4MNH Initiative as well as the presence of various different reproductive health intervention programs and pilots throughout Malawi led the research team to decide on the 16 control sites within the current intervention districts as the most feasible option.

### Study components

To better conceptualize and operationalize the various elements of our mixed methods impact evaluation design, we distinguished the overall work in three study components. The first study component focuses on all quantitative aspects of quality of care based on data collected at the facility level. The second study component focuses on quantitative aspects of health service utilization based on data collected at the community level. A third cross-cutting qualitative study component complements the two quantitative components, in line with the overall mixed methods design described above. We purposely focus on multiple quantitative and qualitative indicators, because we expect that a complex health system intervention, such as a PBF coupled with CCT, will produce multiple effects. We do not expect change to be homogenous across all indicators, but rather expect that while the intervention may be successful to produce change on some dimensions of utilization and quality, it may fail to do so on other dimensions. This discrepancy is not per se problematic, but needs to be reported to adequately inform policy makers on the changes which can be expected from the application of a combined PBF and CCT intervention. The two quantitative study components keep the conceptual division of our research as outlined in the theoretical framework (see Figure 1). The qualitative study component in contrast follows an emerging pattern rooted in the grounded theory approach [43] in response to the preliminary findings yielded by the two quantitative study components. Final interpretation of results will rely on the joint appraisal of the findings stemming from the quantitative and qualitative study components.

### Study component 1

The first study component relates to the quantitative assessment of service quality and relies on five data collection activities: a) an *infrastructural assessment* at the health facility level; b) a series of *systematic observations of provider patient encounters* for selected ANC and delivery services; c) a *systematic review of patient records*; d) a series of *provider interviews* among maternity staff; and e) a series of *patient exit interviews* conducted at point of exit after ANC, delivery, and PNC service use.

Each of the five data collection activities covers a number of quantitative indicators that follow our research aims, the conceptual framework, and the theory of change described earlier. These quantitative indicators can be divided into four thematic groups: a) *infrastructure indicators* measuring the availability, accessibility, and functionality of facility structures, medications, clinical equipment, and human resources in respect to EmONC, ANC, and PNC; b) *process indicators* measuring the adequacy of technical and interpersonal clinical performance in respect to EmONC, ANC, and PNC; c) *output indicators* measuring the quality of immediate service deliverables related to EmONC, ANC, and PNC; and d) *perception indicators* measuring providers’ and clients’ experience related to aspects of EmONC, ANC, and PNC service delivery and service utilization. Table 1 provides an overview of Study Component 1 including the relevant facility-based data sources, the data collection instruments, and the key quality of care measures.

**Table 1:** Overview quality of care study component

**Study component**	**Data collection activity *(Data source)***	**Tool used**	**Key outcome measures**
***Quality of Care*: Structural & Input elements**	Assessment of facility infrastructure *(health facility, heads of services)*	Structured observation survey	• Adequacy of infrastructural and organizational set-up in relation to EmOC standards
			• Availability, accessibility, functionality of materials and equipment in obstetric care, ANC, PNC services
			• Availability, accessibility of clinical guidelines and protocols related to obstetric care, ANC, PNC
			• Availability, accessibility of essential drugs related to obstetric care, ANC, PNC
	Assessment of providers’ professional qualification & technical knowledge *(maternity care providers)*	Structured interview survey (using clinical vignettes)	• Number & type of provider training activities
			• Level of providers’ technical knowledge on EmOC, ANC, PNC
***Quality of Care*: Process & Outcome elements**	Assessment of provider-patient encounters *(obstetric care visits, ANC visits)*	Structured observation survey	• Clinical case management (clinical assessment, diagnosis, treatment) of obstetric, pregnant, and newborn patients
	Assessment of health facility records *(maternity registers, patient charts)*	Structured observation survey	• Timely identification of obstetric/neonatal complication (patient assessment, diagnostic procedures)
			• Timely supportive and definitive management of obstetric/neonatal complications (intravenous fluids, oxygen, antibiotics, blood transfusion, C-sections)
			• Case outcomes (length of stay, fatality, disability)
***Quality of Care*: Experiential elements**	Assessment of providers’ workload, motivation, satisfaction *(maternity care providers)*	Structured interview survey	• Provider’s role, responsibility, workload
			• Provider’s training background and appreciation
			• Provider’s compensation and incentives
			• Provider’s satisfaction and motivation
	Assessment of clients’ perception, satisfaction, experience *(women attending obstetric care, ANC, or PNC services)*	Structured exit interview survey	• Clients’ demographic and socioeconomic information
			• Type of care received at healthcare facility
			• Type of care received at outside formal health sector
			• Clients’ perception of healthcare services received
			• Clients’ knowledge retention related to danger signs
			• Clients’ satisfaction of healthcare services received

EmOC = Emergency Obstetric Care, ANC = Antenatal Care, PNC = Postnatal Care, QUAN = quantitative, qual = qualitative.

For each of the five data collection activities, we developed *ad hoc* data collection instruments. All data collection instruments in this study component were designed to collect quantitative data based on the quality of care indicators outlined above.

To conduct the infrastructural health facility assessments we developed a *structured checklist* to collect information on building structure, human resources, essential medicines and equipment in line with national guidelines and international recommendations regarding the provision of EmONC and ANC. The checklists are designed to not only identify the availability, but also the level of functionality and accessibility of technical equipment and the overall service organization.

A different set of *structured checklists* was developed for the systematic observation of provider-patient encounters during obstetric and ANC service provision. These clinical care checklists collect infromation on the level of quality of routine service processes during patient encounters. There are different checklists for both routine obstetric and routine ANC service activities to identify how clinical tests, medical procedures, patient interview topics, and prescribed drugs adhere to current national treatment standards for each of the examined services.

A third type of *structured checklists* was developed to conduct the review of patient records. These checklists are structured to systematically extract clinical documentation form facility-stored patient charts to retrospectively assess the quality of care that has been delivered in patients with obstetric complications (i.e. pre-eclampsia, eclampsia, hemorrhage, prolonged or obstructed labor, neonatal asphyxia). These checklists are designed to identify the extent to which clinical performance (i.e. procedures, interventions, and treatment options) adheres to national guidelines. The information collected by this systematic chart review differs in so far from the information obtained by the direct clinical care observations in that we try to capture quality of care aspects directly relevant to obstetric emergency cases. This focus on the management of obstetric complications is otherwise not possible with direct clinical observations, due to the relatively small numbers that can feasibly be observed.

To conduct provider interviews, we developed a *structured questionnaire* to assess the effect of RBF4MNH incentives on working conditions, motivation and satisfaction of facility-based maternity staff. In addition, this provider survey instrument is designed to also identify objective information on providers’ prerequisites for technical aspects of quality of care, such as training and clinical knowledge levels, by using clinical vignettes [44]. These vignettes are developed to replicate clinical scenarios common to obstetric, ANC, PNC service delivery and reflect classic presentations of health complaints together with pertinent clinical findings. All vignettes are tailored to the epidemiological profile of Malawi and are aligned with national care protocols.

Another set of *structured questionnaires* was developed to conduct exit interviews with patients seen at the obstetric, ANC, and PNC services of each facility. These questionnaires collect information on how service quality is perceived by those using them, to further determine aspects of client satisfaction with the way services are delivered. Clients’ views on quality will complement the quality of care information obtained through the infrastructural, clinical, and record review checklists.

Sampling techniques differ for each of the data collection activities listed above. Most sampling frames included in this study component consist of small overall figures, such as the total number of health facilities (33) and the total number of maternity care providers (approximately 1–2 for each of the 29 rural facility clusters and 6–10 in the 4 second-level facilities).

For the infrastructural facility assessments and the structured provider surveys, either facility level or maternal care provider level is considered as entry point for sampling. For these two data collection activities, full samples are used in order to include all available study units or study subjects. The sampling approach for the direct clinical observations needs to take into account the actual occurrence of observed events (deliveries might not occur on a daily basis, ANC clinics take place only at certain days in the week), the variation in length of some of these events (deliveries might need to be observed over multiple hours), the variations in willingness of subjects to consent (i.e. providers and patient involved in a case), and the available number and length of stay of interviewer teams at each of the facilities. For this reasons, and in line with previous research [45,38], we expect each data collection team to spend three consecutive days per each health facility. All cases encountered during this time period which consent to participate in the observations are included.

For the client exit interviews we expect the same limitations in case frequency with women exiting obstetric services after childbirth as outlined for the direct observations. Sampling of participants of obstetric care exit interviews therefore follows the same sampling techniques as indicated above. As ANC and PNC services are only provided during specific week-days at most health facilities, we expect higher numbers of patients attending these services within a confined time period. For client interviews of women exiting ANC and PNC services we therefore anticipate to obtain systematic random samples of service users at each facility during these clinic days.

The sampling approach for the patient record review follows sample size estimations found in the literature [46] and is based on the number of indicators used in the checklist (Table 1). Thus, the target samples include 30 maternal chart reviews per facility in order to reach a total sample size of approximately 900 reviews. Medical records are selected following a two-stage sampling procedure. First, based on the case-logs kept in each facility’s maternity unit all cases diagnosed with obstetric complications, prolonged admissions, or fatal outcomes within the preceding three months are identified. For each eligible case identifying information (i.e. date of presentation, patient name, medical registration number) is separately listed by the review team. Second, out of these generated lists random samples of 30 patient cases are drawn and their medical records retrieved from the facility’s medical record office based on the identifying information.

### Study component 2

The second study component relies on a *population-based household survey* to provide a quantitative assessment of utilization patterns across the entire spectrum of maternal care services. In line with our overall conceptual framework and specific research objectives, the survey collects information on women’s use of maternal care services (ANC, delivery, and PNC, including family planning) and the out-of-pocket expenditure incurred in the process of seeking care. In addition, in order to allow for a more scrupulous evaluation of factors associated with health service utilization and out-of-pocket expenditure, the survey gathers information on the household’s socio-demographic and economic profile. This is especially important given our intention to measure the distributional impact of the intervention among socioeconomically different client groups in relation to both utilization and out-of-pocket spending. The quantitative indicators in this component can be divided into two main groups: a) *demand indicators* measuring various determinants of maternal care utilization during and after pregnancy among women living in the EmONC catchment areas; and b) *socioeconomic indicators* measuring numerous determinants of income, property, and social status of women and their households to allow for assessment of equity aspects in service utilization among households in the EmONC catchment areas. Table 2 provides an overview of Study Component 2 including the relevant household-based data sources, the data collection instrument used, and the key access and utilization outcome measures.

**Table 2:** Overview service utilization study component

**Study component**	**Data collection activity *(Data source)***	**Tool used**	**Key outcome measures**
***Utilization*: Health-seeking elements**	Assessment of demand for maternal care services *(women)*	Structured interview survey	• Proportion of women in catchment areas with facility-based deliveries
			• Proportion of women in catchment areas with four ANC visits during pregnancy.
			• Proportion of mothers in catchment areas with first ANC visit during first pregnancy trimester
			• Proportion of women and newborns in catchment areas with at least one PNC visit
***Utilization*: Livelihood asset elements**	Assessment of household socioeconomic status *(women)*	Structured interview survey	• Equity in distribution of facility-based deliveries among households in catchment areas
			• Equity in distribution of number and timing of ANC visits among households in catchment areas
			• Equity in distribution of number and timing of PNC visits among households in catchment area

ANC = Antenatal Care, PNC = Postnatal Care, QUAN = quantitative, qual = qualitative.

The household survey targets exclusively households where at least one woman has completed a pregnancy in the prior twelve months. To identify the women, we apply a three-stage cluster sampling procedure [47]. First, we define clusters; then within cluster we identify relevant Enumeration Areas (EA); and then, within each EA, we identify households that meet our selection criteria (i.e. having at least one woman who has completed a pregnancy in the prior twelve months). In line with the RBF4MNH intervention and with the overall health system structure, we define clusters as the EmONC catchment area of the 33 facilities present in the four districts. Within the cluster, we opted to use EAs rather than villages as initial starting point, because we could not retrieve complete information on the villages contained within a given EmONC catchment area. The limited number of clusters represents an important constraining factor in relation to sample size calculations. Assuming an intra-cluster correlation coefficient of 0.04 and a power of 0.8, a sample of 1800 women allows us to identify a significant impact on the primary study outcome (i.e. utilization of facility-based delivery) if the increase between baseline and endpoint is at least 20%. A sample of a total of 1800 households entails interviewing approximately 25 to 28 women in each EA.

The analytical approach for quantitative Study Components 1 and 2 is identical. Data analysis will rely on the computation of a difference-in-differences (DID) model for each of the outcomes of interest. In line with the overall conceptual model, this yields multiple estimates with the expectation that the intervention may induce change on some indicators, but not on others. The DID approach represents the most suitable analytical model the absence of randomization, as it allows us to systematically estimate the extent to which intervention and control groups mature differently over time. Different maturation over time in measured variables can thus be identified across the three data collection time points, while the resulting true effect estimates can be compared to the defined counterfactual [48,49].

To estimate the true causal effect of the intervention (RBF4MNH) on the multiple quality of care and health service utilization measures between baseline, mid-term, and end line, the DID assumes parallel trends in both intervention and control sites in indicators aside from those caused by the intervention. In situations where the parallel trend assumption is not fully given, incorrect estimation of the true effect will result. In our case, effect estimation is strengthened by the fact that the analytical model relies on multiple post-test measurements, at mid-term and endline, rather than a simple before-and-after design as in many other impact evaluations. Multiple post-test measurements allow for a more precise estimation of the effect as the observable trend in change due to the intervention can be better identified, minimizing the risk that the observed change is simply the product of a secular trend [50-52].

### Study component 3

The third study component refers to the qualitative assessment of both quality of care and service utilization. As mentioned earlier in the text and in line with the overall mixed methods explanatory design [41], the qualitative component relies on a grounded theory approach [43], in which qualitative information is coded, compared and re-categorized as new themes or issues emerge.

This study component consists of qualitative data collection activities and relies on a mixture of *non-participant observations*, *in-depth interviews*, and *Focus Group Discussions* (FGDs) with both users and providers of maternity care services [53]. An additional set of *key informant interviews* with policy and implementing stakeholders is planned to shed light on the overall socio-political context that characterized the introduction of the intervention to allow the research team to further understand observed results. As mentioned earlier, qualitative data collection activities are largely intended to explain the heterogeneity we expect to observe across facilities and communities in relation to all the outcomes observed quantitatively. This is considered to lead to a more comprehensive understanding of the underlying cause-effect relationships, compared to what would otherwise be possible through an exclusive use of quantitative methods. In other words, the qualitative component is shaped in a way to fill the knowledge gaps identified by the quantitative components [41].

Sampling approaches for the qualitative study component differ from those in the quantitative components. Both users and providers of healthcare services are sampled purposely to ensure the theoretical relevance of the selected samples in relation to the themes to be explored [53]. To the extent possible given financial and pragmatic constraints, qualitative data collection continues until saturation and redundancy are reached [54,55]. In line with the grounded theory principle, sampling techniques are geared towards an emerging design, allowing for the inclusion of new constituencies of respondents should new important relevant themes emerge as we progress through qualitative data collection [43].

While the specific themes to be explored will be defined only once the preliminary quantitative analysis is completed, more general attention is given to exploring the experience of providing and receiving care under the new service purchasing scheme. Providers are probed to reflect on if and how the RBF4MNH intervention has contributed to improve their working conditions, to increase their motivation, and to enable them to provide quality services to their communities. In addition, users and potential users of care (i.e. women in need of maternal care services, but not using them) are probed to reflect on if and how the RBF4MNH intervention in the experience of the communities has facilitated equal financial access to maternity services and has improved the quality of these services. While a comprehensive process evaluation assessing the fidelity of implementation [56,57] is not possible given the financial resources at our disposal, the use of non-participatory observation as well as the interviews with providers and communities planned within the framework of our impact evaluation nevertheless allow us to identify potential gaps in the implementation process and to understand how such gaps relate to the observed effects.

All interviews and FGDs are conducted in the local languages by trained research assistants working under the direct supervision of the research team. All verbal material (interviews and FGDs) is tape-recorded, fully transcribed, and translated into English for analysis. Transcripts and translations are checked for content consistency and accuracy. For quality assurance reasons, all non-participant observations are carried out by members of the research team with specific training in taking accurate memos that will later serve as formal material for the overall process analysis. Analysis of the qualitative information is carried out with support of the software NVivo [58]. In line with our underlying grounded theory approach, qualitative analysis will rely on an inductive standard comparison method [43]. The analysis begins with a first reading of the memos and transcripts to acquire familiarity with the data. Categories and sub-categories are developed, modified and extended on the basis of what themes emerge as the analysis proceeds. Links between categories are identified to illuminate the understanding of the research question. Analyst triangulation is applied across all qualitative data sets. At least two independent researchers conduct the analysis separately and only compare and contrast their findings at a later stage. An additional valuable source of triangulation is provided by comparing findings across data sources (interviews, FGDs, and observations) and across respondents (policy stakeholders, providers, and users). When needed, the research team will refer back to the quantitative analysis to elucidate understanding of the emerging qualitative findings and vice versa. As indicated earlier in the description of the overall mixed methods approach, the final result interpretation and the subsequent policy recommendations emerge once both analytical processes are completed and quantitative and qualitative findings are brought together.

### Ethical approval

The study protocol, comprehensive of all of its quantitative and qualitative tools, received ethical approval by the Ethics Committee of Faculty of Medicine of the University of Heidelberg, Germany. In addition, the single study components and their specific quantitative and qualitative tools (including interviews and clinical chart reviews) underwent ethical approval by the College of Medicine Research and Ethic Committee (COMREG), the ethical board located at the College of Medicine, Malawi. Prior to ethical approval by the University of Heidelberg, the study protocol underwent a multidisciplinary competitive peer review process coordinated by the United States Agency for International Development (USAID) Translating Research Into Action (TRAction) Project and its awardee, the University Research Company (URC), and was approved for funding under Subagreement No. FY12-G01-6990. The qualitative component of our study protocol is reported in conformity with the Qualitative Research Review Guidelines (RATS), required in a BMC publication.

## Discussion

Impact evaluations serve multiple purposes: to create empirical evidence, to advise project management, to guide policy decisions, and to inform budget allocations [59]. The recent promotion of PBI schemes represents a good example of how an innovative policy in health systems development – although scientifically backed-up by only meager evidence – receives a lot of attention from implementing organizations, national health policy makers, and international funding agencies [60,61]. To tap into existing knowledge and to generate stronger evidence are the goals of this impact evaluation. To provide comprehensive and robust results that fill at least some of the identified knowledge gaps in the PBI literature, our study design follows closely the implementation process of a PBI scheme. Our choice for a mixed methods design rests on the awareness that understanding the processes through which health interventions produce change is as important as measuring the actual change produced. It follows that a comprehensive assessment of health interventions can therefore only be achieved by coupling quantitative methods with qualitative ones as part of a systematic mixed-methods study design [62]. As guiding principle for impact evaluation studies, the *International Initiative for Impact Evaluation* (3ie) proposes evaluation research to not only focus on aspects that seem to work, but also to supply explanations on the why (or why not) [63]. In this understanding, impact evaluation designs have to focus on both outcome and processes by considering each of the following six key elements: 1) the underlying *causal chain*; 2) *context*-related factors; 3) anticipation of *impact heterogeneity*; 4) a credible *counterfactual*; 5) *counterfactual and factual analysis* of results; and 6) the use of *mixed methods*.

Our approach to impact evaluation explicitly attempts to address all these six elements. To start with, to unravel the underlying causal chain, we adopt a theory-based approach to impact evaluation [64]. We do this through the development of a theory of change closely linked to the initial conceptual framework which, as indicated in the literature, serves both to guide the initial research aims and to inform the development of the study design. To increase its robustness, the initial conceptual framework defining the concepts of utilization and quality is rooted in existing literature [37,39], but was later integrated into one single theory of change which allows us to merge and map all possible causal chains related to the introduction of the RBF4MNH intervention. During this theoretical process, it was important to us to also accommodate specific issues related to the Malawian context that potentially reflect the different factors that determine the causal chains leading to service quality and utilizations, such as the extreme shortage of skilled health care providers, recurring stock-outs for essential medicines and equipment, the poorly developed referral system, the large portion of rural population, the under-funded user fee exemption policy, or the serious economic crisis the nation has been facing for years [65].

Understanding the context as an essential part of the evaluation approach, we allow contextual elements to enter our design on multiple levels. First, in our theory of change, we explicitly consider how social, political, cultural, and economical contextual elements may interfere with the intervention to produce both intended and unintended effects. Second, on a thematic level, some contextual determinants are subject to those research questions (e.g. satisfaction with working environment or perception of service quality) that directly aim at evaluating relationship and interaction between financial incentives and provider motivation or access inequities. Third, on a methodological level, the use of an explanatory mixed methods design provides the opportunity to purposefully investigate contextual elements that might have been overseen initially. For instance, the mixed methods design explicitly allows to explore and incorporate information on how and why social, economic, and cultural factors shape the success or failure of provider-targeted performance incentives, especially with regards to molding intrinsically and extrinsically motivated behavior [66,67]. To gain deeper understanding of healthcare worker motivation it thus not only of importance in relation to quality service delivery, but also allows to shed more light on health worker attrition and retention in general, as this is of special importance within the Malawian context with its extreme human resource for health crisis and the recently implemented policies to counteract this situation [68]. Similarly, the application of a mixed methods design allows us to unravel the contextual factors surrounding the implementation of the CCT and their potential effect on increasing equity or inequity [69]. Not only for our study purpose, but also in the broader frame of Malawi’s current poverty reduction strategies, a deeper understanding of such contextual factors modulating utilization and accessibility of reproductive health services will be of value to a variety of national population development programs [70].

We expect impact heterogeneity to emerge in our study in relation to two factors. First, we expect the intervention to produce different effects across the various concerned facilities. In line with what is stated in the paragraph above, we are aware that specific contextual elements in each district and in each EmONC catchment area will interact with the intervention to produce differential effects. We are aware that since the selection of the facilities receiving the intervention was non-random, chances that heterogeneous effects will be observed are even higher. Again, we return to the application of a mixed methods design as a tool to understand heterogeneity, unraveling key success as well as key failure factors. Second, as outlined in the methods section, we do not expect the intervention to produce uniform results across all selected indicators. It would be naïve to imagine that the intervention could only work to produce exactly and exclusively the initially expected results. Unlike prior evaluations, which focused on a restricted number of indicators closely aligned with the ones set by the program itself [18,20], we explicitly chose to monitor a broader range of services with the aim of capturing unexpected effects. The comprehensive nature of our evaluation, including both providers (at multiple levels of care) and users, while targeting multiple outcome indicators, is therefore more likely to yield heterogeneous results. Thus, we are more likely to capture both successes and failures of the intervention. The challenge, but also an additional opportunity, ahead is the appraisal of heterogeneity across outcome measures in the light of the complementary qualitative data. In combination, this yields more robust evidence as to what changes the intervention is able to induce or not, which is facilitated by triangulation across the multiple data sources available within the study [53].

Counterfactual-based analysis requires reference to a comparison group that allows to estimate how the outcome measure would have changed in the treated population in absence of the intervention [42]. Identifying a proper counterfactual is essential to estimate the cause-effect relationship, i.e. being able to attribute the observed change to the intervention under study [71,72]. Identifying a robust counterfactual for the quantitative study components was the most challenging aspect of our impact evaluation design. The team was left to follow the intervention design, with no option to propose randomization (potentially yielding a trial) or allocation of the intervention exclusively to the facilities above a given quality score (potentially yielding a regression-discontinuity design) [51]. The pre- and post-test design, allowing for the application of DID modelling techniques, was the only feasible option. While we are aware that a fully experimental design would have been preferable from a scientific point of view, we are confident that the DID analytical model will allow for sufficiently accurate estimation of the effect while controlling for history and maturation bias [42], thanks also to the application of multiple post-test measures. At the same time, simply following the intervention team’s plans and aligning our design with their decisions gained us the respect and support of those implementing the program.

We opted to select controls located within the districts for two primary reasons. First, this is considered preferable from a scientific point of view, since the expectation is that facilities (and catchment communities) within a same district are more likely to be similar than facilities (and catchment communities) across districts. This limits the risks that underlying contextual differences are responsible for the observed changes, rather than the intervention per se. Second, it was impossible to select control sites beyond the district boundaries and to be sure that these control sites would not be affected by another maternal and neonatal health program in the very near future, making it impossible for us to attribute causal effects. Still, choosing as controls facilities and catchment communities located within the same districts introduces the risk of a potential spill-over effect from the intervention areas [73], while any contamination of intervention sites due to proximity to non-intervention sites may lead to under-estimation of our effect measurements. In addition, close proximity between interventions and control areas might bias responses or behavior of both clients and providers in the control areas. Still, we feel confident that given the multiple sources of data available within the study, we will be able to control for potential bias by relying on an extensive triangulation process at the analytical stage of our work.

In conclusion, the study protocol presented here is an example for a rigorous mixed methods impact evaluation design that addresses all characteristics desirable for theory-based impact evaluation research. Building the impact evaluation on an explanatory mixed methods design offers the opportunity to address our research aims and conceptual framework comprehensively, especially since our outcome measures – healthcare quality and service utilization – require a multi-dimensional approach. The operationalization of our research design in three components – a quantitative quality of care, a quantitative service utilization, and a qualitative cross-cutting study component – follows the thematic, but also the conceptual structure of the study. As the aim of this impact evaluation is to generate evidence on performance incentives, we feel confident that this explanatory mixed methods design will be able to contribute to existing evidence by addressing the current knowledge gaps related to the effect of output-based financing models, but will also generates specific information relevant for the Malawian health policy context.

## Competing interests

The authors declare that they have no competing interests.

## Authors’ contributions

All authors participated in the conceptualization of the study, the development of the research objectives and relevant theory of change. MDA developed the overall mixed methods design, with contribution from PJR, AM, TBo, and SB. MDA, PJR, and TBa were responsible for the quantitative design. MDA and MS were responsible for the qualitative design. SB developed the quality of care study component and all related indicators with contributions from AM and MS. MDA and PJR developed the health service utilization component and all related indicators with contributions from DM. SB and MDA drafted the manuscript with contribution from all authors. All authors read and approved the final manuscript.

## Pre-publication history

The pre-publication history for this paper can be accessed here:

http://www.biomedcentral.com/1472-6963/14/180/prepub
